# Is there a link between pre-existing antibodies acquired due to childhood vaccinations or past infections and COVID-19? A case control study

**DOI:** 10.7717/peerj.10910

**Published:** 2021-02-09

**Authors:** Bilge Sumbul, Hilmi Erdem Sumbul, Ramazan Azim Okyay, Erdinç Gülümsek, Ahmet Rıza Şahin, Baris Boral, Burhan Fatih Koçyiğit, Mostafa Alfishawy, Jeffrey Gold, ALİ Muhittin Tasdogan

**Affiliations:** 1 Department of Medical Microbiology, Bezmialem Vakıf University, Faculty of Medicine, Istanbul, Turkey; 2 Department of Internal Medicine, University of Health Sciences, Adana Health Practice and Research Center, Adana, Turkey; 3 Department of Public Health, Kahramanmaraş Sütçü İmam University, Faculty of Medicine, Kahramanmaraş, Turkey; 4 Department of Infectious Diseases and Clinical Microbiology, Kahramanmaraş Sütçü İmam University, Faculty of Medicine, Kahramanmaraş, Turkey; 5 Department of Microbiology, University of Health Sciences, Adana Health Practice and Research Center, Adana, Turkey; 6 Department of Physical Medicine and Rehabilitation, Kahramanmaraş Sütçü İmam University, Faculty of Medicine, Kahramanmaraş, Turkey; 7 Infectious Diseases Consultants and Academic Researchers of Egypt (IDCARE), Cairo, Egypt; 8 World Organization, Watkinsville, GA, USA; 9 Department of Anesthesiology and Reanimation, Hasan Kalyoncu University, Faculty of Health Sciences, Gaziantep, Turkey

**Keywords:** COVID-19, Cross-protection, Antibody titers, Pandemic

## Abstract

**Background:**

There is growing evidence indicating that children are less affected from COVID-19. Some authors speculate that childhood vaccinations may provide some cross-protection against COVID-19. In this study, our aim was to compare the circulating antibody titers for multiple childhood vaccine antigens, as an indicator of the state of immune memory between patients with COVID-19 and healthy controls, with a specific aim to identify the association between disease severity and antibody titrations which may indicate a protective function related to vaccine or disease induced memory.

**Methods:**

This study is a case-control study including 53 patients with COVID-19 and 40 healthy volunteers. COVID-19 severity was divided into three groups: asymptomatic, mild and severe. We measured the same set of antibody titers for vaccine antigens, and a set of biochemical and infection markers, in both the case and control groups.

**Results:**

Rubella (*p* = 0.003), pneumococcus (*p* = 0.002), and *Bordetella pertussis* (*p* < 0.0001) titers were found to be significantly lower in the case group than the control group. There was a significant decline in pneumococcus titers with severity of disease (*p* = 0.021) and a significant association with disease severity for *Bordetella pertussis* titers (*p* = 0.014) among COVID patients. Levels of AST, procalcitonin, ferritin and D-dimer significantly increased with the disease severity.

**Discussion:**

Our study supports the hypothesis that pre-existing immune memory, as monitored using circulating antibodies, acquired from childhood vaccinations, or past infections confer some protection against COVID-19. Randomized controlled studies are needed to support a definitive conclusion.

## Introduction

The whole world is struggling to control the 2019 Coronavirus Disease (COVID-19), and facing unacceptable mortality in the hundreds of thousands in many countries (Dong, Du & Gardner, 2020). Worldwide development of specific vaccines is underway. Even though some are being approved today, it will take many months for them to be produced and distributed to all susceptible people across the world. This window without a clear pathway to protecting those at-risk mandates scientists seek other effective measures to mitigate the severity of disease and reduce the future death toll.

Given the available data indicating that children are generally less likely to be affected by COVID-19, and that those between 6 and 10 years of age have shown significantly lower mortality and less severe disease (Dong et al., 2020) leads one wonder if the compulsory child vaccination programs practiced in many countries have a role in mitigating COVID disease in that age group. Further, it poses the question, can one or more of these vaccines act as a vehicle for preventing, or modulating, the severity of COVID-19?

In Turkey, where this study was conducted, an expanded childhood immunization program is being implemented by the Ministry of Health. According to the estimates by the World Health Organization and UNICEF, the coverage of basic childhood vaccines, such as Bacillus Calmette-Guerin (BCG), Tetanus-diphtheria-pertussis, Polio, Measles, Rubella, Hepatitis B, *Haemophilus influenzae* B and Pneumococcal conjugate vaccines currently range between 88% and 99% of children in the country (World Health Organization & UNICEF, 2020).

Our group was the first to hypothesize that childhood vaccines provide some level of cross-protection against COVID-19 (Gold, 2020; Okyay et al., 2020). Soon afterwards, several other authors proposed similar hypotheses (Salman & Salem, 2020; Lyu et al., 2020). We proposed that one or more childhood vaccines, or comparable antibodies from a past infections, may indicate a network of immune memory that can be reactivated to provide some type of cross-protection. It may be adaptive elements, such as the antibodies themselves which recognize coronavirus to some level, or it may be the product of bystander activation by weakly recognized antigens that initiate reactivation of memory clones which produce cytokines to drive the process of “trained innate immunity” through tissue level priming of the local response network.

The enhancement of innate immune function by priming activation, more focused response and better regulation has been recognized for a number of vaccines (Netea et al., 2016). The primary goal, and major benefit of vaccines is to provide either circulating available effector activity against a specific agent, or a pool of memory cells capable of being activated to provide rapid and lasting protection. A side benefit of many vaccines, particularly live agent vaccines, is the development of lasting trained innate immunity.

In many places in the world scientists are currently testing the BCG vaccine for its ability to provide a level of protection against COVID-19. They postulated the role of the BCG vaccine based on prior research that showed the ability of BCG to protect against unrelated infections. Also, epidemiological studies have shown an increase in COVID-19 prevalence in areas with lower vaccine use (Miller et al., 2020).

Measles vaccine was also proposed as a possible mitigator of COVID-19 disease. Similarities were noted in their cutaneous manifestations (Recalcati, 2020). Further, the measles vaccine has been used as a vector for other coronavirus specific vaccines including Severe Acute Respiratory Syndrome (SARS) and the Middle East Respiratory Syndrome Coronavirus (MERS-CoV). These vaccines were able to induce multifunctional T cell response in a mouse model (Bodmer et al., 2018).

Studies are needed to further test these hypotheses. We hypothesized that there might be a relationship between the levels of circulating antibodies we can measure, that we believe reflect the memory pools acquired following childhood vaccinations, or past infections, and COVID-19 disease. Therefore, in this study, our purpose was to measure antibody titers in patients with COVID-19, and a matched pool of healthy controls from the same community. This was done with the specific aim of measuring the association between disease severity and antibody titers as predictors of protection in COVID-19 disease development.

## Materials and Methods

This study, planned as a case-control study, was conducted at the Adana City Training and Research Hospital, Internal Medicine and COVID Clinic. Written informed consent of patients and volunteers were obtained, between April 1, 2020 and May 1, 2020. During the study, 53 patients with COVID-19 without co-morbidity, over the age of 18, and 40 healthy volunteers were included in the study.

### Study population

Patients who were diagnosed by their histories, physical examinations, imaging and laboratory findings, and who agreed to enroll in the study, were included. Patients with diabetes mellitus, thyroid disease, hyperlipidemia, kidney failure, heart disease, hematological disease, lung disease, rheumatic disease, presence of malignancy, pregnancy and those who did not agree to participate in the study were excluded. After all patients and healthy volunteers were included in the study, a detailed history was taken, a physical examination was performed, and the age and sex of participants were recorded. The case group was divided into three subgroups according to the severity of the disease: asymptomatic, mild and severe COVID-19 cases.

### Laboratory testing

Both oropharyngeal and nasopharyngeal swabs were collected for each patient. The swabs were taken to the laboratory and processed using rapid extraction kits. The samples were assessed using a Roche LightCycler Instrument (Device reference number: 05815916001, Serial number: 11927; Roche, Basel, Switzerland). They were handled and processed in accordance with cold chain storage conditions. In addition to routine blood tests for the patients and healthy volunteers included in the study, antibody titers were assessed. For antibody titers, 5 cc blood samples were taken from the patients, the serum part was separated by centrifugation for 10 min at 2,000 Revolutions Per Minute and placed in Eppendorf tubes and stored at −80 °C degrees. The hs-Troponin levels of the patients and healthy volunteers included in the study was measured with the UniCel DXI Analyzer (Beckman Coulter, Brea, CA, USA). Sodium (Na), potassium (K), calcium (Ca), glucose, alanine aminotransferase (ALT), aspartate aminotransferase (AST), blood urea nitrogen (BUN), creatinine, procalcitonin, sedimentation rate, C reactive protein (CRP), fibrinogen, troponin, ferritin, D-dimer and whole blood cell levels were measured using an automated Aeroset chemistry analyzer (Abbott, Plymouth, MN, USA) with appropriate Abbott commercial kits.

### Pre-existing antibody titers

Pre-existing antibodies to be measured were determined based on the vaccines commonly used in childhood. Serum levels of Measles Virus IgG, Rubella Virus IgG, Mumps Virus IgG, Diphtheria IgG, *Bordetella pertussis* IgG, Tetanus IgG, *Haemophilus influenzae* B IgG, were evaluated using Serion ELISA classic (InstitutVirion\Serion GmbH Friedrich-Bergius-Ring 19 97076 Würzburg, Germany) brand commercial kit. Serum levels of Varicella-Zoster Virus IgG and Pneumococcus IgG were analyzed using the Testline (TestLine Clinical Diagnostics Krizikova 68612 Brno Czech Republic) brand kit. The titers of antibodies were determined using purified antigens.

Levels of ≥200, ≥20, ≥100, ≥1, ≥0.35, ≥50, ≥0.50, ≥1.15, and ≥15 antibody units were considered as positive for measles, rubella, mumps, pneumococcus, diphtheria, *Bordetella pertussis*, tetanus, varicella and *Haemophilus influenzae* B, respectively.

### Statistical analysis

Data were expressed as values, percentages and medians (minimum-maximum). In dichotomous variables, the difference between case and control was tested using the Pearson’s chi-square test. The difference between quantitative parameters between case and control was evaluated using the Mann Whitney U test. Disease severity was divided into three groups as asymptomatic, mild and severe. The difference of quantitative parameters among these three groups was evaluated with the Kruskal Wallis test. Post-hoc analysis of Kruskal Wallis test was performed using Dunn test for significant results and false discovery rates were also presented as Benjamini–Hochberg Adjusted *p* values. Spearman correlation analysis was used to determine whether there was a correlation between antibody titers and other measurement-related biological or biochemical parameters.

### Ethical considerations

The study was performed according to the tenets of the Declaration of Helsinki for research involving human subjects. The study was approved by the Ethics Committee of Çukurova University with the date of May 8, 2020 and number 52/99. The study was also approved by the Ministry of Health. Written consent was obtained from the participants before starting the study.

## Results

The median age of the case group included in the study was 42 (min = 21, max = 87), and the median age of the control group was 51.5 (min = 25, max = 78). The age distributions of both groups were similar (*p* = 0.186). The gender distribution of the case and control groups were also similar (*p* = 0.694), 52.5% of the case group, and 56.6% of the control group were male.

When comparing the case and control groups in terms of certain biological or biochemical parameters, it was found that median Na, Ca, troponin, ferritin levels and lymphocyte and platelet counts were significantly lower in the COVID-19 group. In contrast the median ALT, AST, procalcitonin, CRP, fibrinogen D-dimer levels and sedimentation rate were significantly higher in the COVID-19 group (Table 1). It should be noted that two female patients had troponin values over the 16 ng/l threshold. None of the controls exceeded this threshold.

**Table 1:** Comparison of clinical parameters between case and control groups.

Biological or biochemical parameters	Case group (Median (min–max))	Control group (Median (min–max))	*p*
**Sodium**	**138.00 (129.00–143.00)**	**140.00 (135.50–143.20)**	**<0.0001**
Potassium	4.29 (3.57–4.92)	4.37 (3.89–5.18)	0.179
**Calcium**	**9.00 (8.30–11.90)**	**9.50 (8.50–11.50)**	**0.013**
Glucose	101.00 (68.00–346.00)	102.00 (75.00–377.00)	0.497
**ALT**	**21.00 (6.00–71.00)**	**17.10 (7.50–62.80)**	**0.007**
**AST**	**28.00 (15.00–86.00)**	**21.50 (12.50–76.60)**	**<0.0001**
Urea	28.00 (11.00–115.00)	32.80 (16.60–55.00)	0.072
Creatinin	0.75 (0.36–2.12)	0.76 (0.46–1.59)	0.776
**Procalcitonin**	**0.05 (0.03–50.00)**	**0.03 (0.01–0.05)**	**<0.0001**
**Sedimentation**	**36.50 (13.00–120.00)**	**6.50 (1.00–11.00)**	**<0.0001**
**C reactive protein**	**13.30 (1.45–223.00)**	**0.40 (0.00–1.35)**	**<0.0001**
**Fibrinogen**	**348.42 (115.00–900.00)**	**236.50 (178.00–344.00)**	**<0.0001**
**Troponin**	**4.00 (1.00–176.00)**	**10.00 (2.00–13.00)**	**0.003**
**Ferritin**	**68.00 (10.00–2714.00)**	**239.00 (167.00–356.00)**	**<0.0001**
**D-dimer**	**472.00 (136.00–6950.00)**	**319.00 (237.00–389.00)**	**<0.0001**
WBC	6.00 (3.20–12.80)	6.00 (3.50–9.10)	0.855
Neutrophile	4.00 (1.50–9.00)	3.25 (2.00–6.20)	0.091
**Lymhocyte**	**1.50 (0.40–2.40)**	**2.10 (1.20–3.80)**	**<0.0001**
Monocyte	0.60 (0.20–1.60)	0.60 (0.30–1.00)	0.987
Eosinophil	0.20 (0.10–0.60)	0.20 (0.00–0.80)	0.261
Hemoglobin	13.50 (8.80–16.00)	13.55 (10.40–16.50)	0.416
**Plateletes**	**205.00 (111.00–393.000)**	**243.00 (95.00–485.00)**	**0.014**

**Notes:**

min: minimum; max: maximum.

The parameters that were statistically significant in the comparison of the groups were indicated with bold.

We evaluated the differences in the antibody titers to the common vaccine antigens between the two groups. All of the participants, both in case and control groups, were positive for measles titers. Of the case group, all but three patients (94.3%), and of the control group all but two people (95.0%), were positive for rubella. All participants, except for one person in control group, were positive for mumps. Of the case group 22 patients (41.5%), and of the control group 30 people (75.0%), were positive for pneumococcus, and the difference between cases and controls was significant (*p* = 0.001). Of the case group 17 patients (32.1%), and of the control group 12 people (30.0%), were positive for diphtheria. Of the case group 10 patients (18.9%), and of the control group 27 people (67.5%), were positive for *Bordetella pertussis*, the difference between cases and controls was significant (*p* < 0.0001). Of the case group, 38 patients (71.7%), and of the control group 21 people (52.5%), were positive for tetanus. Of the case group 49 patients (92.5%), and of the control group 35 people (87.5%), were positive for Varicella. Of the case group 48 patients (90.6%), and of the control group 35 people (87.5%), were positive for *Haemophilus influenzae* B.

When antibody titers were compared between case and control groups, the Rubella, pneumococcus and *Bordetella pertussis* titers were found significantly lower in the case group than the control group (Table 2).

**Table 2:** Comparison of pre-existing antibody titers between case and control groups.

Antibody titers	Case group (Median (min–max))	Control group (Median (min–max))	*p*
Measles Ig G titers	9,632.50 (263.60–453,221.00)	6,919.75 (201.60–38,133.00)	0.207
Rubella Ig G titers	**95.10 (1.30–300.90)**	**159.30 (17.80–1,833.40)**	**0.003**
Mumps Ig G titers	1,194.00 (291.40–9,445.30)	986.90 (62.20–4,177.30)	0.251
Pneumococcus Ig G titers	**0.79 (0.16–9.53)**	**2.30 (0.08–10.03)**	**0.002**
DiphtheriaIg G titers	0.23 (0.01–1.38)	0.19 (0.03–1.77)	0.895
B. PertussisIg G titers	**20.50 (4.50–511.20)**	**78.35 (1.70–693.50)**	**<0.0001**
TetanusIg G titers	1.20 (0.00–9.70)	0.55 (0.00–24.30)	0.489
Varicella Ig G titers	2.95 (0.66–6.28)	2.92 (0.47–5.77)	0.813
H. influenza BIg G titers	68.40 (5.00–1,522.60)	51.55 (7.00–407.50)	0.055

**Notes:**

min: minimum; max: maximum.

The parameters that were statistically significant in the comparison of the groups were indicated with bold.

The case group was divided into three groups according to the severity of the disease: asymptomatic, mild and severe COVID-19 cases. The antibody titers and certain biological or biochemical parameters were compared in these severity groups. The distribution of titers for pneumococcus and *Bordetella pertussis* were significantly different with the severity of the disease. Pneumococcus titers decreased in a clear fashion as the disease became more severe. However, the difference in *Bordetella pertussis* titers was not clearly indicative of the severity of disease. Rather, the titer was quite low among those with mild disease impacting the finding of significance (Table 3). The levels of AST, procalcitonin, ferritin and D-dimer appeared to increase with disease severity (Table 4). Post-hoc analysis for results found significant after the comparison of the aforementioned three groups and false discovery rates were also presented in Tables 5 and 6.

**Table 3:** Comparison of pre-existing antibody titers in terms of disease severity.

Antibody titers	Asymptomatic cases (Median (min–max))	Mild cases (Median (min–max))	Severe cases (Median (min–max))	*p*
Measles Ig G titers	3,996.90 (263.60–39,643.00)	10,577.00 (684.00–453,211.00)	6,762.60 (1,462.80–38,254.00)	0.314
Rubella Ig G titers	79.15 (29.50–300.90)	108.20 (1.30–217.50)	95.05 (29.00–202.20)	0.897
Mumps Ig G titers	960.80 (402.80–6,060.30)	1,252.30 (291.40–9,445.30)	1,213.40 (361.00–3493.80)	0.820
**Pneumococcus Ig G titers**	**2.01 (0.21–7.38)**	**0.50 (0.17–9.53)**	**0.45 (0.16–1.28)**	**0.021**
DiphtheriaIg G titers	0.38 (0.03–1.22)	0.18 (0.02–1.38)	0.18 (0.01–0.97)	0.551
**B. Pertussis Ig G titers**	**37.95 (12.90–85.30)**	**17.00 (4.50–511.20)**	**36.15 (12.80–66.70)**	**0.014**
TetanusIg G titers	1.65 (0.00–5.40)	1.10 (0.00–9.70)	1.40 (0.00–6.30)	0.740
Varicella Ig G titers	3.84 (1.34–6.28)	2.92 (1.10–5.09)	2.57 (0.66–4.86)	0.237
H. influenza BIg G titers	62.25 (17.30–299.00)	70.30 (5.00–965.60)	53.50 (9.40–1,522.60)	0.993

**Notes:**

min: minimum; max: maximum.

The parameters that were statistically significant in the comparison of the groups were indicated with bold.

**Table 4:** Comparison of clinical parameters in terms of disease severity.

Biological or biochemical parameters	Asymptomatic cases (Median (min–max))	Mild cases (Median (min–max))	Severe cases (Median (min–max))	*p*
Sodium	138.00 (136.00–141.00)	138.00 (129.00–142.00)	137.50 (132.00–143.00)	0.593
Potassium	4.21 (3.87–4.61)	4.31 (3.57–4.79)	4.36 (3.64–4.92)	0.616
Calcium	8.90 (8.30–9.80)	9.10 (8.40–10.00)	9.05 (8.30–11.90)	0.420
Glucose	102.50 (79.00–221.00)	99.00 (68.00–346.00)	104.50 (96.00–153.00)	0.470
ALT	20.00 (11.00–38.00)	21.00 (6.00–65.00)	36.50 (16.00–71.00)	0.237
**AST**	**24.00 (20.00–32.00)**	**25.00 (15.00–86.00)**	**44.00 (33.00–73.00)**	**<0.0001**
Urea	27.00 (16.00–43.00)	27.00 (12.00–115.00)	35.00 (11.00–44.00)	0.428
Creatinin	0.72 (0.40–1.10)	0.74 (0.36–2.12)	0.78 (0.62–1.05)	0.579
**Procalcitonin**	**0.05 (0.03–0.10)**	**0.05 (0.03–2.00)**	**12.00 (2.00–50.00)**	**<0.0001**
Sedimentation	29.00 (19.00–65.00)	38.50 (13.00–120.00)	79.00 (23.00–111.00)	0.117
C reactive protein	4.94 (1.49–58.40)	14.10 (1.45–195.00)	77.70 (2.50–223.00)	0.068
Fibrinogen	316.66 (199.60–378.89)	358.66 (167.28–694.00)	507.66 (115.00–900.00)	0.388
Troponin	3.00 (2.00–8.00)	4.00 (1.00–176.00)	6.50 (2.00–22.00)	0.245
**Ferritin**	**29.35 (10.00–111.60)**	**104.30 (16.30–1,420.10)**	**365.00 (33.20–2,714.00)**	**0.022**
**D-dimer**	**338.50 (136.00–1,740.00)**	**440.00 (160.00–6,650.00)**	**1,134.00 (472.00–3,760.00)**	**0.017**
WBC	5.65 (3.20–7.30)	6.10 (3.20–10.40)	6.80 (3.30–12.80)	0.333
Neutrophile	3.30 (1.50–5.10)	3.90 (1.70–8.70)	4.55 (2.10–9.00)	0.354
Lymhocyte	1.40 (0.80–2.00)	1.60 (0.40–2.30)	1.10 (0.90–2.40)	0.561
Monocyte	0.60 (0.50–0.80)	0.60 (0.30–1.00)	0.50 (0.30–1.60)	0.887
Eosinophil	0.30 (0.10–0.60)	0.20 (0.10–0.60)	0.15 (0.10–0.30)	0.330
Hemoglobin	13.30 (11.00–15.80)	13.60 (10.20–16.00)	13.00 (8.80–15.50)	0.771
Plateletes	183.50 (135.00–313.00)	213.00 (127.00–393.00)	198.00 (111.00–380.00)	0.566

**Notes:**

min: minimum; max: maximum.

The parameters that were statistically significant in the comparison of the groups were indicated with bold.

**Table 5:** Post-hoc analysis of significant results observed in biochemical parameters when comparing disease severity groups.

Biochemical parameters	Asymptomatic cases-Mild cases (1–2)		Asymptomatic cases-Severe cases (1–3)		Mild cases-Severe cases (2–3)		KW^c^
	*p*^a^	FDR_BH_^b^	*p*^a^	FDR_BH_	*p*^a^	FDR_BH_	*p*
**AST**	0.145	0.145	**0.0001**	**0.0003**	**0.001**	**0.0015**	**<0.0001**
**Procalcitonin**	0.743	0.743	**0.001**	**0.0015**	**0.0001**	**0.0003**	**<0.0001**
**Ferritin**	**0.010**	**0.03**	**0.023**	**0.00345**	0.598	0.598	**0.022**
**D-dimer**	0.341	0.341	**0.006**	**0.018**	**0.015**	**0.0225**	**0.017**

**Notes:**

a *p* value obtained with Dunn Test.

b Benjamini–Hochberg Adjusted *p* value.

c Kruskall Wallis test.

The parameters that were statistically significant were indicated with bold.

**Table 6:** Post-hoc analysis of significant results observed in antibody titers when comparing disease severity groups.

Antibody titers	Asymptomatic cases-Mild cases (1–2)		Asymptomatic cases-Severe cases (1–3)		Mild cases-Severe cases (2–3)		KW^c^
	*p*^a^	FDR_BH_^b^	*p*^a^	FDR_BH_	*p*^a^	FDR_BH_	*p*
**Pneumococcus Ig G titers**	**0.017**	**0.025**	**0.011**	**0.033**	0.374	0.374	**0.021**
**B. Pertussis Ig G titers**	**0.006**	**0.018**	0.459	0.459	0.109	0.163	**0.014**

**Notes:**

a *p* value obtained with Dunn Test.

b Benjamini–Hochberg Adjusted *p* value.

c Kruskall Wallis test.

The parameters that were statistically significant were indicated with bold.

We observed significant correlations between the antibody titers and biological or biochemical parameters in the COVID-19 group. The mumps titers showed a positive correlation with lymphocyte count (Rho = 0.375, *p* = 0.006). The rubella titers showed a negative correlation with the level of Ca (Rho = −0.353, *p* = 0.015). The pneumococcal titers showed a negative correlation with the level of Ca (Rho = −0.296, *p* = 0.043). The *Bordetella pertussis* titers showed a negative correlation with the sedimentation rate (Rho = −0.498, *p* = 0.001) and with the CRP levels (Rho = −0.389, *p* = 0.010). The tetanus titers showed a negative correlation with the level of D-dimer (Rho = −0.312, *p* = 0.023). The varicella titers showed a negative correlation with the level of ALT (Rho = −0.384, *p* = 0.005), the level of AST (Rho = −0.405, *p* = 0.003), procalcitonin (Rho = −0.319, *p* = 0.048), the sedimentation rate (Rho = −0.404, *p* = 0.008) and the level of CRP (Rho = −0.378, *p* = 0.012). The *H. influenzae* B titers showed a positive correlation with the level of Na (Rho = 0.402, *p* = 0.003).

## Discussion

In this study, we compared a set of randomly chosen healthy controls with a cohort of COVID-19 patients being seen at the same institution and representing the same community. We compared these populations with respect to a number of standard biochemical markers and a rather large set of antibody titers representing common childhood vaccines, or the disease they are designed to provide protection against. This was done to attempt to establish some correlational information that would aid the diagnostic efficacy and prognostic accuracy for COVID-19. We also investigated whether circulating antibodies acquired due to childhood vaccinations, or past infections, appear to be related to a protective effect that is involved in mitigating the severity of the disease.

We found that median sodium and calcium levels were lower in the COVID-19 group than the controls. In a meta-analysis of disease biochemistry, it was reported that COVID-19 patients have lower than normal sodium levels. Further, it was speculated that low body sodium may have resulted in the overexpression of ACE-2. This would increase the binding efficacy of SARS CoV-2 to cells and result in higher disease susceptibility (Luo, Li & Dai, 2020). Di Filippo et al. (2020) found that hypocalcemia is highly prevalent in COVID-19 patients. We believe that the assessment of sodium and calcium levels may be of some assistance in diagnosis of COVID-19.

Median troponin levels were found lower in the COVID-19 case group in this study. Yet we observed a progressive increase in troponin from asymptomatic to severe cases of COVID-19. This was not, however, statistically significant. Also, two female COVID-19 patients (3.7% of all patients) had troponin over the 16 ng/l threshold, while none exceeded this level in control group. There is evidence in the literature that SARS-CoV-2 may cause myocardial injury in some patients (Li et al., 2020; Long et al., 2020). Huang et al. (2020) reported 12% of patients having elevated levels of troponin. Although we found a lower frequency in our study than Huang et al. (2020) our findings support the observation of myocardial damage in some COVID-19 patients.

We found that median ferritin levels were lower in the COVID-19 group than the controls. However severe COVID-19 patients had higher median ferritin levels than did the mild or asymptomatic cases, or the controls (See Tables 1 and 4). Mehta et al. (2020) reported high ferritin levels in severe COVID-19 patients and indicated that high ferritin levels may be associated with fatal hypercytokinaemia and the subsequent multiorgan failure. We concluded from previous work and the confirmation from our study that high ferritin levels may be a predictor of poor prognosis in COVID-19.

Lymphocyte and platelet counts were significantly lower in the COVID-19 group. Lymphopenia is a common finding in COVID-19. It has been reported to be associated with severe disease (Terpos et al., 2020). We also observed the lowest median lymphocyte count in severe COVID-19 patients. Xu, Zhou & Xu (2020) suggested that SARS CoV-2 may reduce platelet production, increase platelet destruction and increase platelet consumption. This would cause thrombocytopenia in COVID-19 patients. Some authors also speculated that thrombocytopenia is associated with severe COVID-19 disease (Terpos et al., 2020; Lippi, Plebani & Henry, 2020). In our study, although we did not find a relationship between disease severity and platelet count, our findings support that platelet levels may be low in patients with COVID-19.

Median ALT, AST, procalcitonin, CRP, fibrinogen D-dimer levels and sedimentation rate were significantly higher in the COVID-19 group than the control group. All of these biological or biochemical markers had their lowest levels in asymptomatic patients and their highest levels in patients with severe disease. For AST, procalcitonin and D-dimer levels a statistically significant change was observed with the increasing severity of disease. Several authors have reported elevated liver aminotransferase levels in COVID-19 patients. Moreover they found it was associated with disease severity (Guan et al., 2020; Wang et al., 2020a, 2020b). These findings indicated that SARS-CoV-2 may infect the liver itself and cause cellular damage. Procalcitonin, CRP and sedimentation rate are all inflammatory markers which are expected to increase in infectious diseases (Aringer, 2020). Velavan & Meyer (2020) in their review, reported studies that find increased procalcitonin and CRP levels in severe COVID-19. Lapić, Rogić & Plebani (2020) reported sedimentation rate is also associated with severe COVID-19. In a meta-analysis increased procalcitonin values were reported to be associated with a 5-fold higher risk of severe COVID-19 (Lippi & Plebani, 2020). We concluded that the increase in procalcitonin particularly indicates that bacterial co-infections are probably concurrent with severe COVID-19. Several studies have shown an increase in fibrinogen and D-dimer levels in COVID-19 patients, similar to our observation (Gao et al., 2020; Panigada et al., 2020; Spiezia et al., 2020). This suggests that coagulopathy is frequent in COVID-19 when low platelet levels are also taken into consideration. We also think that the aforementioned biological or biochemical markers may have prognostic values in COVID-19.

Our main hypothesis was to test whether pre-existing antibody titers confer protection against COVID-19. Rubella, pneumococcus and *Bordetella pertussis* titers were found to be significantly lower in the COVID-19 group than the control group. The distribution of titers of pneumococcus and *Bordetella pertussis* significantly differed according to the severity of the disease. Pneumococcus titers linearly decreased with the severity of the disease. *Bordetella pertussis* titers, however, were found to be higher in both asymptomatic and severe cases of COVID-19 and much higher in the controls, but lower in mild cases (Tables 2 and 3). Franklin et al. (2020) reported that SARS-CoV-2 spike glycoproteins share structural similarities with the fusion proteins of both measles and mumps viruses and they found 29% amino acid sequence homology between the macro domains of SARS-CoV-2 and rubella; and concluded that measles, mumps, rubella (MMR) vaccination could improve the outcome of COVID-19 infection. Similarly, Gold reported that MMR vaccine appears to confers protection from COVID-19 in a study of the vaccination coverage in different countries around the world and their incidence of COVID-19 disease (Gold, 2020). Although we found a significant difference in COVID-19 and control groups in terms of rubella titers, we did not find a significant relationship in the severity of COVID-19 disease based on rubella titer.

There is evidence of pneumococcus and *Bordetella pertussis* co-infection in COVID-19 patients (Kozak et al., 2020; Zhu et al., 2020). Zhu et al. (2020) reported that 59.5% of COVID-19 patients were infected with pneumococcus. Further a study published in September, 2020 reported a potential cross-reactivity between SARS-CoV-2 proteins and the pneumococcal proteins (Root-Bernstein, 2020). This may explain the lower pneumococcus titers in severe COVID-19 patients found in our study. We found no relation with *Bordetella pertussis* titer levels and the severity of the disease. However, the rate of individuals with positive IgG was significantly lower in the COVID-19 group as compared to the controls.

With our findings, we cannot definitively say that a certain type of antibody interacts with SARS-CoV-2 and creates a neutralizing effect and cross protection. The antibody levels found in our study only show traces of previous infections or active immunization by vaccination. Our findings may be interpreted with the concept of trained immunity as an alternative mechanism of protection. Trained immunity can be described as “an innate immune memory program induced by certain infections or vaccinations” (Netea & Van der Meer, 2017). Although a recent study has found a relationship between MMR vaccination and COVID-19 severity using mumps titers as a proxy (Gold et al., 2020), it was not possible to verify this with our study, since we used alternate methodology. Live pertussis vaccine has been reported to provide non-specific immune responses in mice models, however this effect waned after the vaccine was inactivated by heat (Cauchi & Locht, 2018). In Turkey, inactive tetanus-diphtheria-pertussis vaccines are given simultaneously. In our study, a significant difference was found with Pertussis antibody titers-, but not with tetanus or diphtheria antibody titers—between the control group and the COVID-19 group. Also, *Bordetella pertussis* titers were higher in asymptomatic and severe cases as compared to mild cases, which indicates that *Bordetella pertussis* antibodies did not directly affect disease severity. This suggests that the presumed protection could possibly be due to a silent Pertussis infection, which may induce trained immunity.

We have also observed some significant correlations with antibody titers with certain biological or biochemical markers. Mumps titers showed a positive correlation with lymphocyte counts. Rubella and Pneumococcus titers showed a negative correlation with calcium levels. *Bordetella pertussis* titers showed negative correlations with sedimentation and CRP levels. Tetanus titers showed a negative correlation with D-dimer levels. Varicella titers showed negative correlations with ALT, AST, procalcitonin, sedimentation and CRP levels. *H. influenzae* B titers showed a positive correlation with sodium levels. These findings demonstrate that multiple different vaccines or past infections may provide some level of protection against COVID-19 either in the form of weakly cross-reactive antibodies, or by providing weakly cross-reactive reactivation of the adaptive memory system and production of cytokines that prime, arm and regulate tissue level innate immunity which retards coronavirus replication and spread in the body with a salutary effect on the development of more severe disease.

Our study has some limitations. The sample sizes of both case and control groups were small. Recruitment of healthy controls was difficult and costly in the pandemic era. Therefore, there was an imbalance in case and control numbers. The titrations of antibody for some of the childhood vaccines commonly delivered (Hepatitis A, Hepatitis B and BCG and polio) were not included in the study. Also, it should be noted that we did not investigate the vaccination history of patients. Thus, the antibodies measured are likely to be due to natural infection as well as vaccination. Nonetheless, to the best of our knowledge, our study was the first to measure pre-existing antibody titers and compare them with healthy individuals. In addition, titers of nine major childhood vaccines were measured in our study.

In conclusion, we presented the biological or biochemical markers that changed in COVID-19 patients as compared to healthy controls. We discussed how these markers change according to the severity of COVID-19 disease. We observed that rubella, *Bordetella pertussis* and pneumococcus titers were lower in COVID-19 patients than in controls. We observed significant correlations between some of the antibody titers and biological or biochemical markers. Our findings support the hypothesis that circulating antibodies indicate some level of protection against COVID-19 that is associated with the memory pool generated by childhood vaccines (or the diseases that they are designed to protect against). Prospective randomized controlled studies are recommended to reach a definitive conclusion.

## Supplemental Information

10.7717/peerj.10910/supp-1 Supplemental Information 1 Raw data.

## Additional Information and Declarations

### Data Availability

The following information was supplied regarding data availability: The raw data are available in a Supplemental File.

### Clinical Trial Registration

The following information was supplied regarding Clinical Trial registration: The study was approved by the Ethics Committee of Çukurova University with the date of May 8, 2020 and number 52/99
